# Femoral tunnel placement in anterior cruciate ligament reconstruction: rationale of the two incision technique

**DOI:** 10.1186/1749-799X-2-10

**Published:** 2007-05-21

**Authors:** Raffaele Garofalo, Biagio Moretti, Cyril Kombot, Lorenzo Moretti, Elyazid Mouhsine

**Affiliations:** 1 Department of clinical methodology and surgical technique, orthopaedics section, University of Bari, Bari, Italy; 2 Department of traumatology and orthopaedic surgery, University Hospital, Lausanne, Swizerland

## Abstract

Endoscopic anterior cruciate ligament (ACL) reconstruction can be performed through one-incision or two-incision technique. The current one-incision endoscopic ACL single bundle reconstruction techniques attempt to perform an isometric repair placing the graft along the roof of the intercondylar notch, anterior and superior to the native ACL insertion. However the ACL isometry is a theoretical condition, and has not stood up to detailed testing and investigation. Moreover this type of reconstruction results in a vertically oriented non-anatomic graft, which is able to control anterior tibial translation but not the rotational component of the instability. Femoral tunnel obliquity has a great effect on rotational stability. To improve the obliquity of graft, an anatomical ACL reconstruction should be attempt. Anatomical insertion of ACL on the femur lies very low in the notch, spreading between 11 and 9–8 o'clock position and the center lies lower than at 11 o'clock position. Femoral aiming devices through the tibial tunnel aim at an isometric placement, and they do not aim at an anatomic position of the graft. Also, a placement of tunnel in a position of 11 o'clock is unable to restore rotational stability. The two-incision technique, with the possibility to position femoral tunnel independently by tibial tunnel, allows us to place femoral tunnel entrance in a position of 10 'clock that can most accurately reproduce the anatomic behaviour of the ACL and can potentially improve the response of the graft to rotatory loads. This positioning results in a more oblique graft placement, avoiding problem related to PCL impingement during knee flexion. Further studies are required to understand if this kind of reconstruction can ameliorate proprioception as well as clinical outcome at a long-term follow-up.

## Background

Endoscopic anterior cruciate ligament (ACL) reconstruction is a surgery that allows most subjects to resume activity at preinjury level. However the estimated failure rate after this surgery remains approximately 10% [1]. For many years the two-incision technique has represented the gold standard operation for ACL reconstruction [2,3]. In the last years, however, the single incision tibial endoscopic technique has been developed to obviate the necessity of the lateral incision and to, potentially, reduce operative time and surgical morbidity.

Many published reports have concluded that there is no difference in subjective, objective, functional, or radiographic mid-terms follow-up outcomes between one or two incision technique [4,5]. However, these studies have not compared the obliquity of femoral tunnels, but nowadays we know that placement of femoral tunnel has a great influence on knee kinematics [6,7]. Transtibial ACL reconstruction has shown some disadvantages in the femoral tunnel placement with respect to the two incision techniques. With transtibial technique, in particular, femoral tunnel can not be placed freely, so this technique dictates a relatively vertical and central non anatomical graft placement compared to the more horizontal and lateral course of the native ACL. This physiometric "central cruciate" cannot control rotational stability and places abnormal force on the knee joint, which could lead to degenerative osteoarthritis in long term [6-8]. To obviate the above problems, drilling femoral tunnel through anteromedial portal has been proposed. With the knee in a maximally flexed position, it seems to be possible to perform a femoral tunnel 5 mm anterior to the posterior capsular insertion through this portal at the 11 o'clock (right) or 1 o'clock position with respect to the apex of the notch [9]. Also the graft placed in this position failed to control rotatotial stability [7]. Recently, a great number of surgeons sustaining the one-incision transtibial reconstruction technique has focused the attention on the double bundle (DB) anatomical reconstruction of the ACL to ameliorate the rotational control on the reconstructed knee [10].

Moreover there are other recognized potential pitfalls of the endoscopic technique, including graft tunnel mismatch [11], interference screw fixation divergence [12] or screw laceration of the graft, posterior cruciate ligament (PCL) impingement [13] and possible violation of posterior cortical wall [5]. The purpose of this paper is first, to describe anatomical single bundle ACL reconstruction with a two-incision technique and second, to discuss the rationale of this technique by reviewing anatomy and biomechanics on femoral tunnel position in ACL reconstruction.

### Surgical technique

#### Setup and graft harvesting

The patient is placed in the supine position with a lateral post just proximal to the knee. In our mind graft choices in primary ACL reconstruction for young persons and sports people is the bone-patellar tendon-bone (BPTB) graft, whereas semitendinosus and gracilis tendons (ST-GR) is reserved for older subjects, women, and those devoted to recreational sports or for patients with some patellar problems.

BPTB is harvested via an incision of 7 cm in average, extending from the inferior pole of patella to the tibial tubercle. The paratenon is divided longitudinally. A central third bone-patella tendon-bone (BPTB) graft is harvested 10 mm wide with 10 or 11-mm × 25-mm tibial bone block and 9 or 10-mm × 20-mm patellar bone block. The block is cut in a trapezoidal fashion at the tibial level and in a triangular fashion at the level of patella to reduce bone stress and anterior knee pain. A small rongeur and a graft shaper are used to trim the graft to the appropriate size. One hole is drilled in each bone block to be used for leading threads. Normally, tibial bone plug is positioned into femoral tunnel and patellar bone plug into the tibial tunnel. A number 2 reabsorbable suture is passed through these holes and used as pulling suture. The tibial bone-tendon junction is marked with a sterile pen to aid in appropriate placement within the femoral tunnel.

Before starting arthroscopic step of reconstruction, patellar tendon defect is closed with a 3.0 reabsorbable suture. We prefer to include the Hoffa tissue in the first proximal stitch. Paratenon is closed with a running suture.

In case in whom we use hamstring tendon the ST-GR tendons are harvested through a 2–3 cm oblique incision made directly over the pes anserinus in line with the hamstring tendons course. Once the sartorius fascia is identified, it is opened and an angled clamp is then used to localize the ST-GR tendon, which is harvested with an open tendon stripper with the limb held in the so-called figure-of-four position. Retained muscle belly is scraped from the tendons and the end of each graft is then sutured for approximately 4 cm with a "Chinese" finger trap stitch of a number 2 nonabsorbable suture. The quadrupled tendon graft is sutured at the looped end using one stitch of a number 1 absorbable suture. The graft is sized and a mark is made at 30 mm from the looped end.

#### Arthroscopic reconstruction

We use a high anterolateral and low anteromedial portal for arthroscopy. A outflow portal is made through the suprapatellar pouch to end in the medial gutter. Articular and meniscal injury are addressed at first. Any remaining fibres of the ruptured ACL are debrided using scissor and motorized 5.5 mm full radius resector. A 10 mm curved rugine is used to debride the medial wall of the lateral condyle just to the posterior capsule so as to identify the site of insertion of native ACL. The same instrument is used to verify that the distance between PCL (posterior cruciate ligament) and medial wall of lateral condyle is at least 10 mm. If it not the case a lateral trochleoplasty is performed.

**Figure 1:**
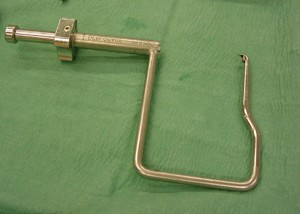
Photograph of the specific rigid femoral drill guide used to create the outside-in femoral tunnel.

**Figure 2:**
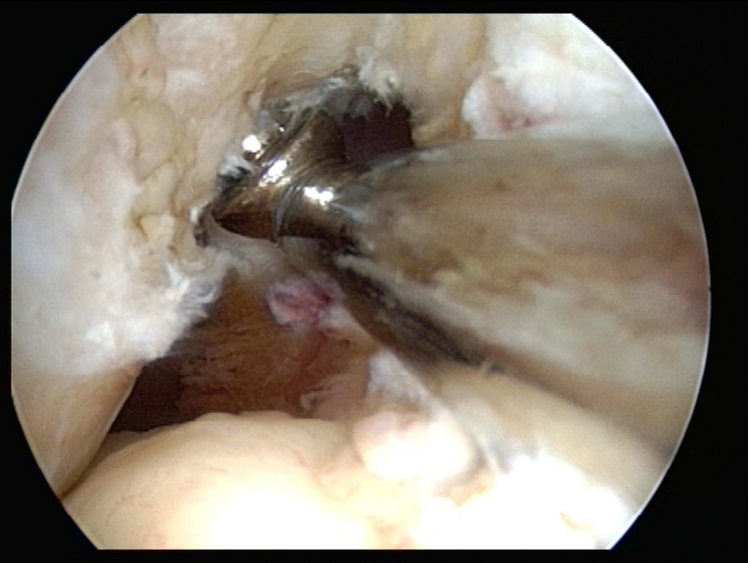
Arthroscopic view of a right knee showing the tip of femoral guide placed in the ACL anatomical footprint lower than roof of the intercondylar notch.

**Figure 3:**
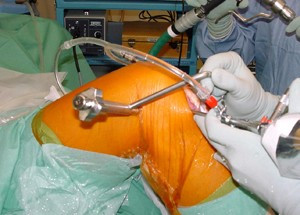
Picture of external view of guide placement on the lateral side of distal part of a right thigh. A little skin incision necessary to perform this technique is showed.

**Figure 4:**
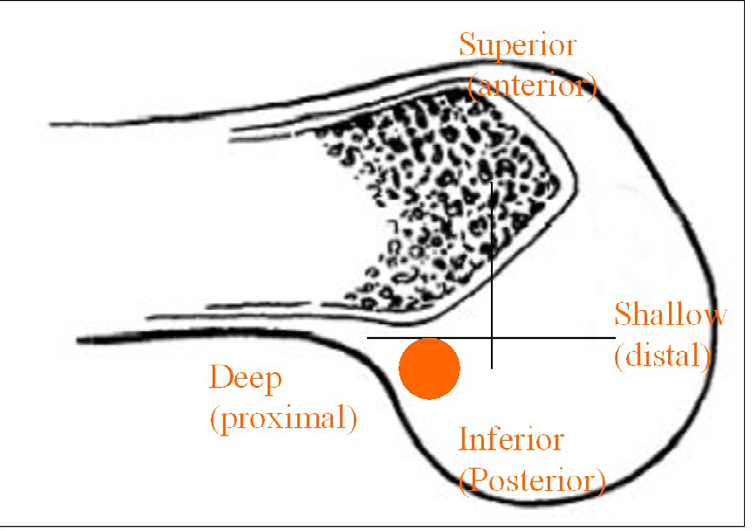
Arthroscopic nomenclature viewing the knee in the sagittal plane, with anatomical nomenclature in parentheses. The circle indicates the site of femoral tunnel to positioning anatomical single bundle reconstruction where the most anterior point of tunnel correspond to isometric point of AM bundle.

With the knee flexed at an average of 90 degree, a specific femoral drill guide (Phusis- Grenoble- France) is inserted through the anteromedial portal (Fig. 1). The tip of the guide is placed immediately behind the footprint of native ACL. The landmarks for a correct placement of guide are the passage between the notch roof and lateral notch wall, and the superior border of cartilage of the posterior part of the lateral femoral condyle. The identification of these key points allows us to place femoral tunnel at 10 o'clock (2 o'clock) at level of native ACL (Fig. 2). The external arm of the femoral guide lies outside on the lateral femoral condyle. A lateral skin incision of an average of 2 cm is made slightly superior to the lateral epicondyle. This incision passed longitudinally through the anterior portion of iliotibial band and is straight to the bone (Fig 3). The guide pin is drilled with a slight oblique direction from back to front and from high to low. An outside-in femoral tunnel of 25 to 30 mm long is established with a cannulated reamer with the diameter identical to the graft. The completed tunnel should have almost no bone in the back edge with the most anterior edge positioned at the level of the isometric point (Fig. 4). The tibial tunnel is created using a 55 degree drilling guide introduced through the anteromedial portal. The tip of the guide is placed slightly medial to the centre of the intercondylar region, 7 mm anterior to the PCL, on a line joining the inner edge of the anterior horn of the lateral meniscus and the medial tibial spine. Drilling is performed in the anteromedial tibia. After the guide pin is placed in a right position, it is overdrilled with a cannulated drill. The size of drill is identical to graft size.

With a suture passer, the graft can be passed into the knee by passing a nylon loop-shuttle suture through the joint. The suture at the end of graft is passed in the loop suture, so the surgeon pulling on the loop suture out of tibial or femoral tunnel, allowing the suture of the graft to go out the tunnel.

Hamstring tendon graft is positioned such that the mark at 30 mm is flushed with the femoral tunnel, whether BPTB is positioned such that the marked tendon-bone junction is flushed with the intra-articular entrance of the femoral tunnel. BPTB is passed with the cortical surface posterior to keep the tendinous portion of the graft as posterior as possible. Different systems of fixation can be used. Anyway femoral fixation is performed at first. A maximal manual tension is applied to the distal sutures of the graft and the knee is cycled through full flexion extension several times for graft pretensioning and settling. The knee is placed in approximately 30 degree of flexion, with one arm of assistant put under the proximal femur, and tibial fixation is carried-out under arthroscopic control. At the end of procedure, the scope is inserted retrograde in the tibial tunnel to verify that during passive knee motion there is no graft motion.

### Rationale of the two incision ACL reconstruction

The native ACL lies in an oblique position with a complex attachment to the femur [14]. This attachment is smaller than tibial, and is semicircular (18 × 10 mm) with a straight anterior border and a convex posterior border. It lies at level of posteromedial wall of the lateral femoral condyle, at the transition between the notch roof a bone cartilage boundary of the posterior part of the lateral femoral condyle. The attachment extends anteriorly 6 to 8 mm from the posterior border which gives the footprint a rounded triangular shape [15,16]. The ACL has a complex anatomy. Many investigators [16,17] have described anatomically separated fibre bundles of the ACL. Based on their tibial attachments, the bundles are called anteromedial (AM) and posterolateral (PL) bundles, some also includes an intermediate bundle [18]. At level of femur, the attachment of AM bundle is anterior and proximal, just behind the top of the intercondylar notch roof. This point is corresponding to the most isometric point [19,20]. Consequently, the most anterior fibers of AM bundle are the most isometric [21,22]. The reason of placing femoral bone tunnel at 11:00 o'clock position for a right knee and 1:00 o'clock for a left knee is related to an attempt to reconstruct the anteromedial bundle. The PL bundle of the ACL represents the bulk of ligament and its fibres are the most posterior and distal and provide stability when the knee is near extension. The femoral attachment site is more important than the tibial attachment because it has a greater effect on the graft length changes as the knee flexes and extends, [21]. In fact, the center of rotation is closer to the femoral attachment than the tibial attachment side, so there is little room for error when placing the femoral tunnel. Clinical results correlate positively with femoral tunnels placed at least 60% posterior to the anterior origin of Blumensaat line in a deep and superior (proximal) position [23].

A cadaveric study, performed by Arnold et al., has confirmed that the anatomical insertion of ACL on the femur lies very low in the notch spreading between 11 and 9–8 o'clock and the center lies lower than at 11 o'clock position [14]. The major part of these fibres lies posteriorly to the isometric point on the medial wall of the femoral condyle [24]. These fibres located behind the most isometric point, are lax during flexion and tight in extension. Chambat has defined the behaviour of these fibres as "favourable non isometry" [25]. During knee extension we can observe a progressive recruitment of the fibres from front (the most isometric) to backward. The "favourable non isometry" is very interesting because knee loading during daily activities occurs at flexion angles of less than 60 degrees, and to reproduce it, an anatomical placement of the graft should be performed during surgery.

The major part of current endoscopic transtibial ACL reconstruction techniques place the graft along the roof of the intercondylar notch, anterior and superior to the native ACL insertion, exposing some of fibers of the non-anatomically placed graft to higher strain rates and risk of failure. Some authors sustaining that a nearly isometric behaviour of the ACL substitute is desirable, with a maximum of 2–3 mm lengthening of the graft towards extension [18,26]. The isometry is widely influenced by femoral tunnel placement. Studies evaluating isometric placement of graft have suggested that the 12-o'clock position with a 2 mm of posterior wall was the most isometric [27], but this position results in a vertically oriented non-anatomic graft [28]. In such situation the anterior stability of the knee is partially controlled, but rotational stability remained uncontrolled, resulting in a persistent pivot shift with consequent pathologic knee kinematics that can be associated with a poorer outcome and long-term arthritis [6,8,29]. Hefzy et al. [21] noted a larger isometric (2 mm) zone superiorly and proximally, so most authors are recommending an entry point high in the notch, at the 11 o'clock position for a right knee with 1 to 2 mm posterior cortical shell, and often this requires the use of a more inferior anteromedial portal resulting in a more demanding technique [30]. However, the current method used to place the femoral tunnel in the 11 o'clock position seems to be inaccurate and moreover analysis of literature shows that the ACL isometry is a theoretical condition, and has not stood up to detailed testing and investigation [22,26,27].

According to cadaveric dissection [14], we create a 25 to 30 mm long femoral tunnel at anatomical insertion site of all, at 10 o'clock position. This positioning is impossible to obtain through a transtibial endoscopic technique [14]. To reach a better femoral tunnel placement the anteromedial portal instead of the transtibial portal with a knee flexed at 130 degrees has been proposed [9]. Moreover, problem about PCL impingement when using transtibial technique should not be ignored. Simmons et al. [13] have showed that placing the femoral tunnel in the coronal plane at 60 degree lowers graft tension in flexion. This minimize PCL impingement of graft. To obtain this positioning with the transtibial technique, a special tibial guide should be used and drilling through the superficial fibers of the medial collateral ligament should be performed [13].

The rationale to perform an anatomical ACL reconstruction is also related to concept of rotational stability. Recent study has shown that placing ACL graft at the 11 o'clock is unable to restore rotational stability [31]. Loh et al [6] in a laboratory robotic study reproduced a single bundle ACL reconstruction positioning the femoral tunnel at 10 and 11 o'clock. They found that the 10 o'clock femoral position restored anterior tibial translation and *in situ *forces towards knee extension significantly better than the 11 o'clock position, and although the resulting knee kinematics was not normal, however, the rotational knee stability was improved. Scopp [7], using a biomechanical model, has shown that reconstructing the femoral tunnel at the oblique anatomic origin of the native ACL, oriented 60 degrees from vertical, more closely restored knee rotational stability than the standard tunnel reconstruction oriented at 30 degrees from vertical [7]. This positioning corresponds to our proposed technique of ACL reconstruction with a "favourable non isometry" [25] and to place the graft in this area, a femoral tunnel at 10 o'clock position on the right lateral femoral condyle should be drilled, with the knee at 90 degrees of flexion.

Recently, the group of surgeon sustaining the one-incision reconstruction technique has shift the attention on the DB ACL reconstruction in attempt to ameliorate the rotational control on the reconstructed knee [32].

Nevertheless, it should be underlined that rotational control of knee is not completely related to ACL. Other peripheral restraints are also responsible for this control, otherwise we could not explain why different people with a complete, subacute ACL disruption show different grades of pivot shift phenomenon. Probably a certain number of ACL disruption are not isolated. However, the evaluation and diagnosis of peripheral associated instability, such as anterolateral rotatory instability is demanding and difficult to assess objectively, so the associated lesions often are not addressed with a consequent persistence of a some rotational instability, that probably could remain using a DB ACL reconstruction.

In our opinion DB ACL reconstruction is a shift of emphasis to an anatomic procedure. Nevertheless, creation of three or four tunnels is technically challenging and some concerns such as theoretical risks of avascular necrosis of the lateral femoral condyle, fracture, graft impingement and difficulty in revision cases should be taken in account. Moreover further clinical trials will be necessary to determine whether these theoretical advantages will translate into superior clinical outcomes.

For instance, according to other authors [28,33,34], anatomical single bundle reconstruction using a two-incision technique seems to be a good technique to position femoral tunnel entrance in a position that most accurately reproduces an anatomic behaviour of the ACL and potentially can improve the response of the graft to rotational loads.

Further biomechanical studies in "vivo" are needed to verify if this positioning of femoral tunnel have some positive effects on long-terms clinical outcome in terms of reduction of pivot shift phenomenon and improved proprioception.
